# Supplementary material for: Overexpression of Orange Gene (OsOr-R115H) Enhances Heat Tolerance and Defense-Related Gene Expression in Rice (Oryza sativa L.)
Source: Genes (Basel). 2021 Nov 26;12(12):1891. doi: 10.3390/genes12121891 (PMC8701904; doi:10.3390/genes12121891)
Supplement: Supplementary file 1 [file genes-12-01891-s001.zip › Supplementary Table S1.pdf]

Supplementary Table S1. Primers used for qRT-PCR analysis in this study. F: forward; R: reverse.

Primer name	Sequence (5' to 3')	Gene ID
OsDREB2A F	AGATTGCTCCGTGCAAGTG	Os04g0572400
OsDREB2A R	CTGGAGCTTCTGGTTTTGCT	
OsDREB1A F	TGGAGCTACTAGAGCTCAATCAACTG	Os01g0165000
OsDREB1A R	TGGCATCGGAAGCCAGAA	
OsAPX2 F	CTCTCCTACGCCGACTTCTAC	Os01g0165000
OsAPX2 R	AGGTGGTCAGAACCTTGTGT	
OsSNAC1 F	GTCAAGACTGATTGGATCATGC	Os03g0815100
OsSNAC1 R	CCAATCATCCAACCTGAGAGA	
OsSOD-Cu/Zn F	GCTCTATTGCGTTGTATGCCA	Os04g0573200
OsSOD-Cu/Zn R	GCTTGACTCCCAAATGGTGAC	
OsLEA3 F	GGCGCAGTACACCAAGGA	Os05g0542500
OsLEA3 R	ACCTGCTCACTCGCCTGT	
OsCATA F	GCCGGATAGACAGGAGAGGT	Os02g0115700
OsCATA R	TCTTCACATGCTTGGCTTCA	
OsCATB F	GGTGGGTTGATGCTCTCTCA	Os06g0727200
OsCATB R	ATTCCTCCTGGCCGATCTAC	
OsP5CS F	TTCATGGGAAAAAATTGGT	Os05g0455500
OsP5CS R	CCGCAATAAGATGCTTGTAC	
NOS F	CAGAGGAGGACGCTGGTGTA	-
NOS R	CCGCTCGAGATCCAGTTAGGAGCTGAAAAC	
Bar F	CGTCAACCACTACATCGAGA	-
Bar R	AAGTCCAGCTGCCAGAAA	
OsOr F	TTTCACTCTTGGTGTGAAGCAGAT	Os02g0651300
OsOr R	GACTTCCTTCACGATTTCATCGTAA	
OsActin F	GGCTGTTTTCCCTAGTATCG	Os03g0718100
OsActin R	GTACGTCCACTGGCATACAG	
TaqMan *Nos* ter F	TTGCGGGACTCTAATCATAAAAA	-
TaqMan *Nos* ter R	GAATCCTGTTGCCGGTCTT	
