# Supplementary figures and images for: Overexpression of Orange Gene (OsOr-R115H) Enhances Heat Tolerance and Defense-Related Gene Expression in Rice (Oryza sativa L.)
Source: Genes (Basel). 2021 Nov 26;12(12):1891. doi: 10.3390/genes12121891 (PMC8701904; doi:10.3390/genes12121891)

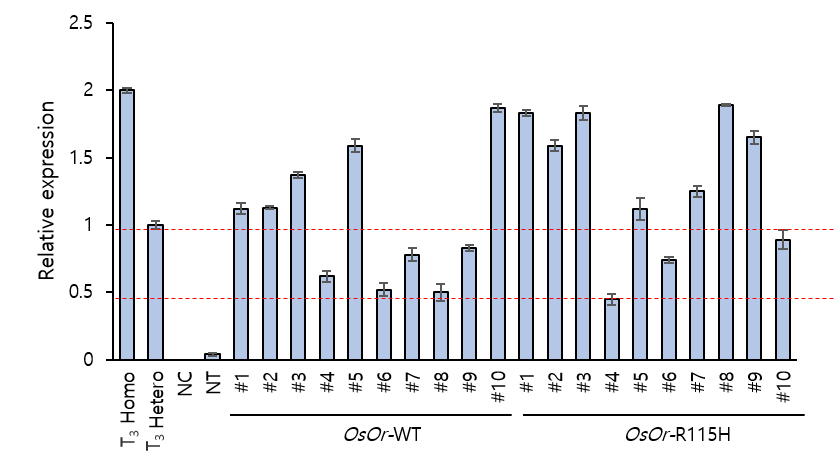

Supplement: Supplementary file 1 [file genes-12-01891-s001.zip › Supplementary Fig S2.tif]

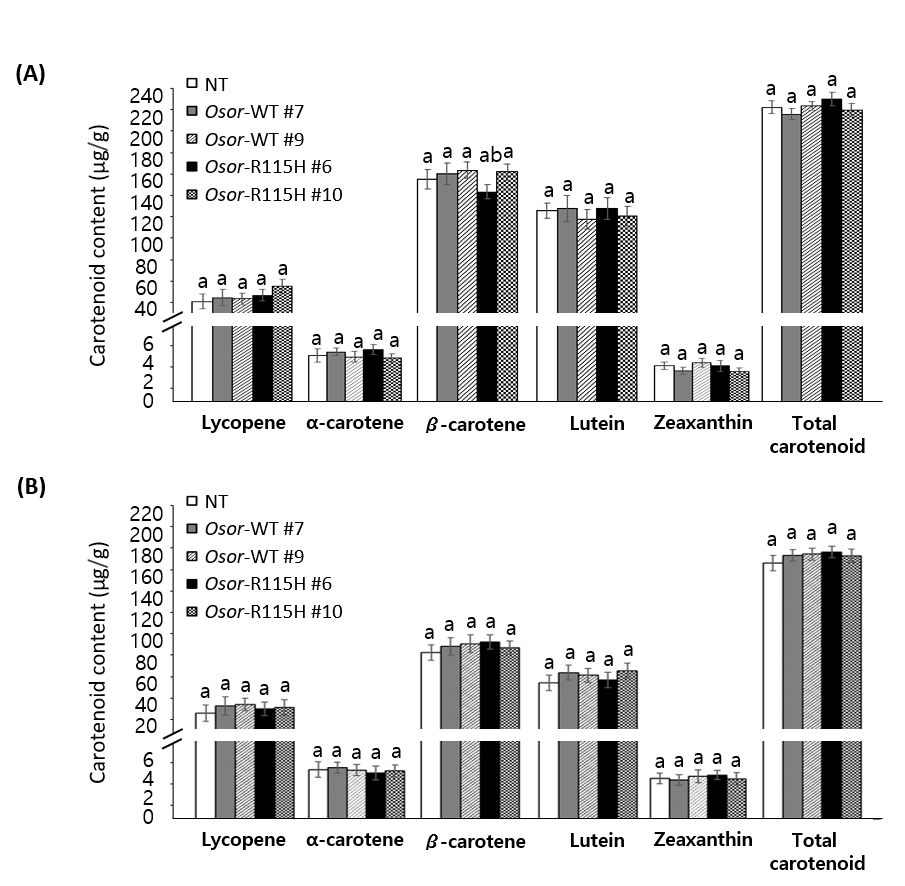

Supplement: Supplementary file 1 [file genes-12-01891-s001.zip › Supplementary Fig S3.tif]

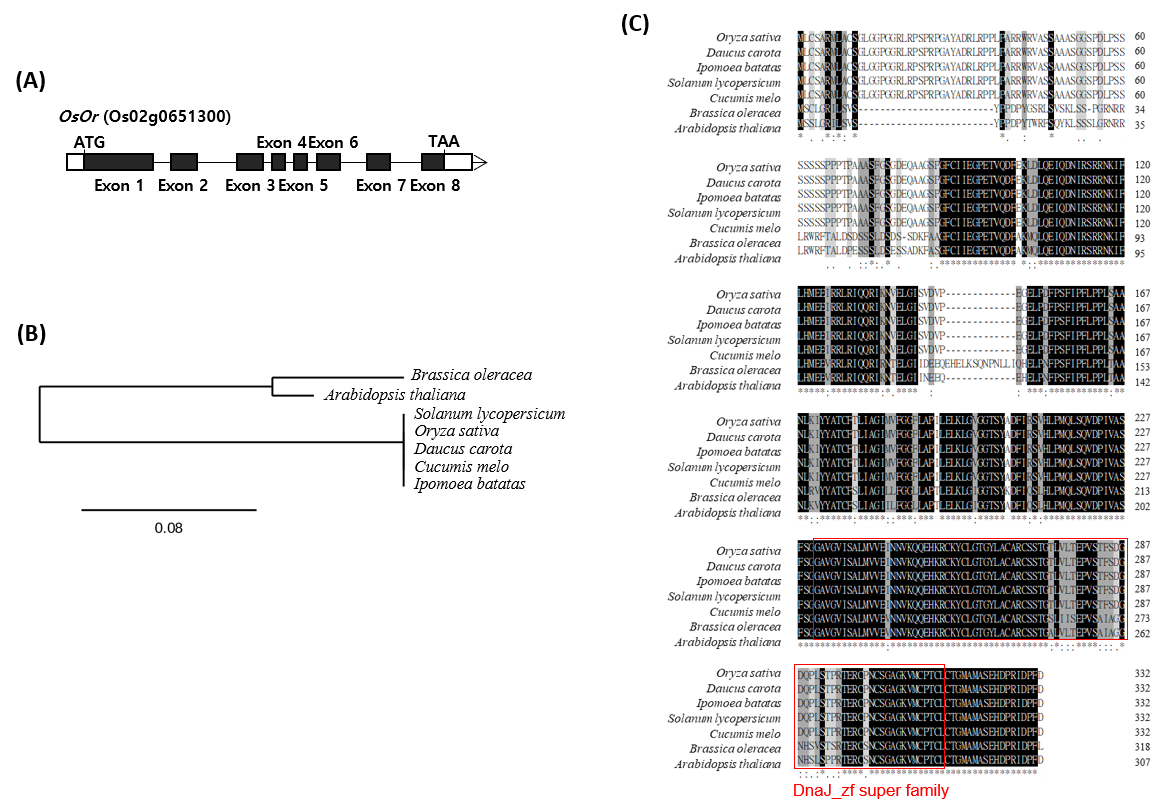

Supplement: Supplementary file 1 [file genes-12-01891-s001.zip › Supplementary Figure S1.tif]
